# Increased Hepato-Splanchnic Vasoconstriction in Diabetics during Regular Hemodialysis

**DOI:** 10.1371/journal.pone.0145411

**Published:** 2015-12-29

**Authors:** Werner Ribitsch, Daniel Schneditz, Casper F. M. Franssen, Gernot Schilcher, Vanessa Stadlbauer, Jörg H. Horina, Alexander R. Rosenkranz

**Affiliations:** 1 Department of Internal Medicine, Clinical Division of Nephrology, Medical University of Graz, Graz, Austria; 2 Institute of Physiology, Medical University of Graz, Graz, Austria; 3 Department of Internal Medicine, Division of Nephrology, University Medical Center Groningen, Groningen, The Netherlands; 4 Department of Internal Medicine, Clinical Division of Gastroenterology and Hepatology, Medical University of Graz, Graz, Austria; The University of Tokyo, JAPAN

## Abstract

**Background and Objectives:**

Ultrafiltration (UF) of excess fluid activates numerous compensatory mechanisms during hemodialysis (HD). The increase of both total peripheral and splanchnic vascular resistance is considered essential in maintaining hemodynamic stability. The aim of this study was to evaluate the extent of UF-induced changes in hepato-splanchnic blood flow and resistance in a group of maintenance HD patients during regular dialysis.

**Design, Setting, Participants, & Measurements:**

Hepato-splanchnic flow resistance index (*RI*) and hepato-splanchnic perfusion index (*QI*) were measured in 12 chronic HD patients using a modified, non-invasive Indocyaningreen (ICG) dilution method. During a midweek dialysis session we determined *RI*, *QI*, ICG disappearance rate (*k*_ICG_), plasma volume (*V*_p_), hematocrit (*Hct*), mean arterial blood pressure (*MAP*) and heart rate (*HR*) at four times in hourly intervals (*t*_1_ to *t*_4_). Dialysis settings were standardized and all patient studies were done in duplicate.

**Results:**

In the whole study group mean UF volume was 1.86 ± 0.46 L, *V*_p_ dropped from 3.65 ± 0.77L at *t_1_* to 3.40 ± 0.78L at *t_4_*, and all patients remained hemodynamically stable. In all patients *RI* significantly increased from 12.40 ± 4.21 mmHg∙s∙m^2^/mL at *t_1_* to 14.94 ± 6.36 mmHg∙s∙m^2^/mL at *t_4_* while *QI* significantly decreased from 0.61 ± 0.22 at *t_1_* to 0.52 ± 0.20 L/min/m^2^ at *t_4_*, indicating active vasoconstriction. In diabetic subjects, however, *RI* was significantly larger than in non-diabetics at all time points. *QI* was lower in diabetic subjects.

**Conclusions:**

In chronic HD-patients hepato-splanchnic blood flow substantially decreases during moderate UF as a result of an active splanchnic vasoconstriction. Our data indicate that diabetic HD-patients are particularly prone to splanchnic ischemia and might therefore have an increased risk for bacterial translocation, endotoxemia and systemic inflammation.

## Introduction

Ultrafiltration (UF) induced hypovolemia activates a variety of compensatory mechanisms to maintain hemodynamic stability during hemodialysis (HD). However, failure of the hemodynamic response to counterbalance central hypovolemia and inadequate plasma refilling may lead to intradialytic hypotension, an acute complication occurring in 15 to 30% of HD treatments [1]. In hypovolemic states an active vasoconstriction of the splanchnic vascular bed increasing both vascular resistance and venous return is considered fundamental to maintain hemodynamic stability [2]. Results from early studies showing a decrease of splanchnic blood flow during HD-treatment are in support of this important compensatory response to hypovolemia [3] [4] [5]. However, it is believed that a pronounced splanchnic hypoperfusion for prolonged periods of time such as during HD and UF might weaken the gut barrier thereby facilitating bacterial translocation, endotoxemia, and systemic inflammation [6] [7] [8]. The adverse consequence of splanchnic hypoperfusion has been termed “gut stunning” [6] in analogy to myocardial stunning observed during HD [9] [10]. Although undisputed in essence, the details are incompletely understood and concise information about the impact of ultrafiltration on hepato-splanchnic vascular resistance and splanchnic blood flow is sparse. Even though a few early studies have addressed some of these questions [3] [4] [5], it is difficult to transfer the results of these studies to a chronic HD-population in a clinical routine setting.

The aim of our study therefore was to investigate the magnitude of ultrafiltration-induced changes in hepato-splanchnic resistance and perfusion in a group of stable end stage kidney disease (ESKD) patients during regular dialysis treatments.

## Material and Methods

### Patients

The study was done in accordance with the Declaration of Helsinki at the Clinical Division of Nephrology, Medical University of Graz, Austria. All patients gave their written informed consent prior to this study, which was approved by the Institutional Review Board of the Medical University of Graz (registration number 23–056 ex 10/11). Subjects with abnormal liver function tests or other hepatic disease were excluded. Patients were studied during their regular midweek treatment with either HD or online hemodiafiltration (HDF) delivered in post-dilution mode. Patients were studied in supine body position and were fasting prior to and during the study period to rule out hemodynamic perturbances of the splanchnic region caused by food intake [11] [12]. The ultrafiltration rate was constant throughout dialysis, the dialysate temperature was set at 37°C, and the dialysate sodium prescription was individualized to match the patient’s plasma sodium concentration as measured at dialysis start. Studies were repeated on the same weekday of the following week.

### Study protocol

Hepato-splanchnic perfusion (*Q*) was measured using a modified indocyanine green (ICG) dilution method as described elsewhere [13] [14]. ICG is a classic dye used for hemodynamic measurements. In plasma ICG binds to albumin and its dilution has been used to examine the magnitude as well as the changes in plasma volume during dialysis as it is not removed from the circulation by the dialyzer [15]. ICG is exclusively eliminated by the liver, also during dialysis. With normal liver function the half-life is about 3 to 4 min so that all dye is removed from the circulation within 15 to 20 min allowing for multiple measurements during the same application. Moreover, the extraction from blood passing the liver sinusoids is almost 100% with normal liver function so that the clearance of ICG refers to total hepato-splanchnic blood flow (*Q*). Four mL of a 5 mg/mL solution of ICG (ICG-PULSION®, PULSION Medical Systems, Munich, Germany) were injected into the venous blood line of the extracorporeal circulation. The first bolus at time *t_1_* (baseline) was delivered within 15 min after treatment start and three subsequent boli were administered in hourly intervals at times *t*_2_, *t*_3_, and *t*_4_. ICG-concentrations were continuously and non-invasively measured by optical means with a sampling period of 20 s (CLI, CritLine® Instrument, Fresenius Medical Care, Utah, USA). Distribution volume and clearance of ICG were derived from analysis of ICG dilution curves assuming single-pool kinetics at the four measuring points *t_1_* to *t_4_* during HD as described elsewhere [14]. The blood disappearance rate (ICG-disappearance rate, the normal range varying between 18–25%/min) was determined from the slope of the elimination curve. Hematocrit (*Hct*) and plasma volume (*V_p_*) were derived from the CLI readings immediately prior to each ICG-injection at times *t*_1_ through *t*_4_.

Hemodynamics were assessed by mean arterial pressure (*MAP*) and heart rate (*HR*). Hepato-splanchnic resistance (*R*) was estimated from the ratio of mean arterial pressure to hepato-splanchnic blood flow (*Q*) assuming negligible hepatic venous pressure. For practical reasons resistance is given in non-SI peripheral resistance units (*PRU*, mmHg.s/mL).

To account for differences in body size, hepato-splanchnic blood flow and resistance were normalized for body surface area (*A*) to obtain hepato-splanchnic flow index (*QI* = *Q/A*, in L/min/m^2^) and hepato-splanchnic resistance index (*RI* = R*A, in *PRU*.m^2^), respectively. Body surface (*A*) was calculated according to the Du Bois formula [16].

### Statistical Analyses

Hemodynamic data obtained in subsequent treatments were averaged to account for repeated measurements in the same subject Results are presented as the mean value ± SD or the median and interquartile range. Normal distribution of the data was verified by the Kolmogorov-Smirnov test. Statistical methods used were two-sample *t*-test for comparison of means and single factor variance analyses with repeated measurement for analyses of temporal changes. The reproducibility of repeated individual measurements was evaluated by Pearson’s correlation coefficient *r* and Wilcoxon signed rank test. A *p* < 0.05 was considered significant. Statistical calculations were done with SPSS, Version 20.0 for Windows (SPSS Inc., Chicago, IL, USA).

## Results

**Fig 1:**
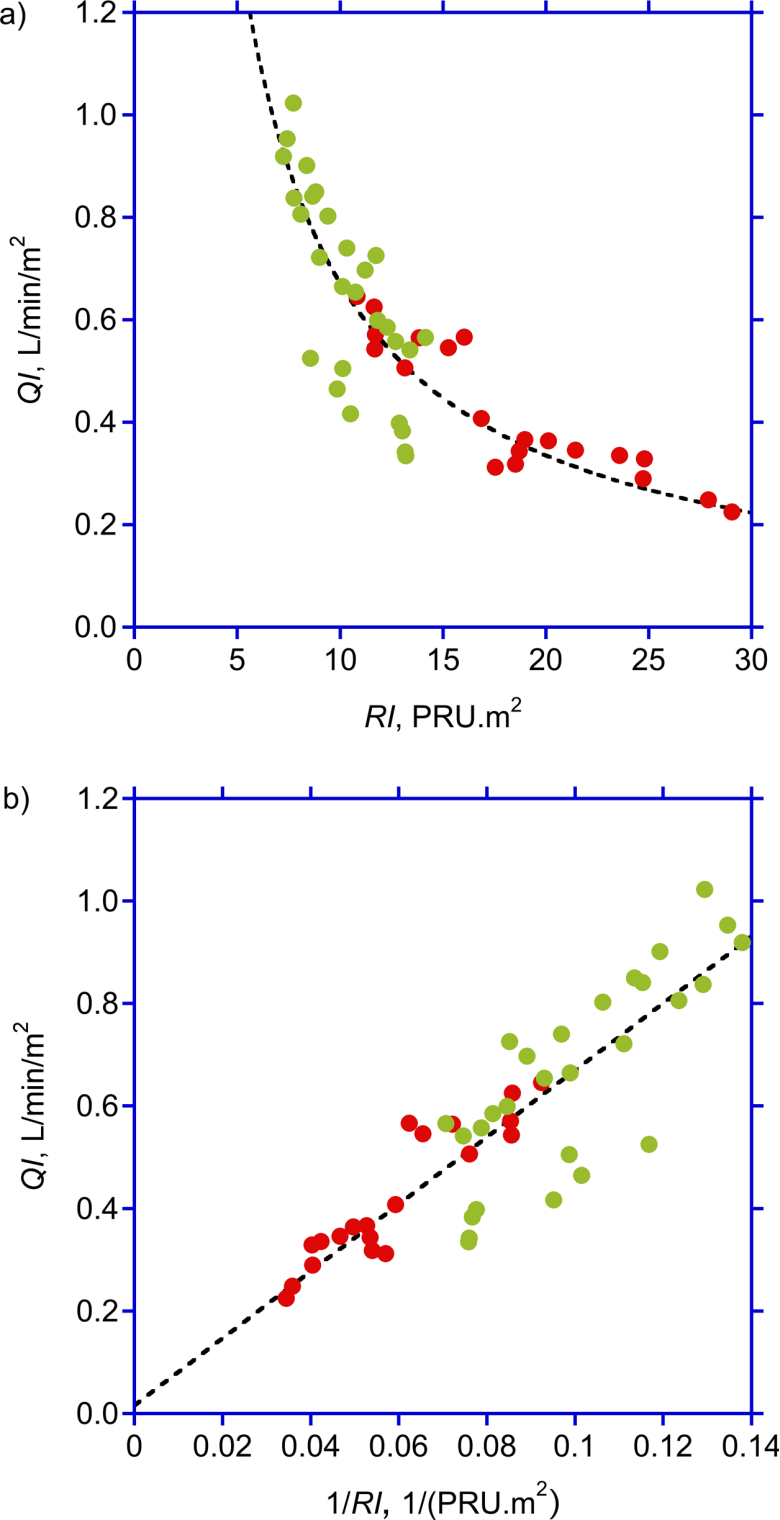
Hepato-splanchnic perfusion and resistance. Splanchnic perfusion index (*QI*) as function of hepato-splanchnic resistance index (*RI*) (top panel) and hepato-splachnic vascular conductance (bottom panel), respectively, during hemodialysis, in diabetic (red symbols) and non-diabetic (green symbols) subjects. Broken lines indicate the best fit of *QI* to *RI* (top panel, *y* = 6.71/*x*, *r* ^2^ = 0.77) and 1/*RI* (bottom panel, *y* = 0.02+6.54*x*, *r* ^2^ = 0.77), respectively.

We studied 12 (6 female) chronic HD-patients in whom ESKD was due to diabetic nephropathy (2 type-I and 1 type-II diabetes mellitus, respectively) (three), focal segmental glomerulosclerosis (one), bilateral nephrectomy due to urothelcarcinoma (one), gestosis (one), hemolytic-uremic syndrome (one), unknown (two), interstitial nephritis (one), hypertensive glomerulosclerosis (one), and amyloidosis secondary to Crohn´s Disesase with ankylosing spondylitis (one). As Crohn’s Disease may affect hepato-splanchnic blood flow depending on disease activity [17, 18], a closer look at the patient history revealed remission as quantified by a Harvey Bradshaw Index Score of 3 (a score < 5 indicating remission, [19]) with leukocyte counts and C-reactive protein levels within the normal range. Relevant extrarenal manifestations of amyloidosis were excluded by echocardiogram and colonic biopsy which were both negative for myocardial infiltrations and colonic amyloid deposits. Therefore, this non-diabetic patient was included in the study. The most frequent comorbidities were hypertension (ten), coronary heart disease (four), peripheral artery disease (three) secondary hyperparathyreoidism (eleven) and renal anemia (twelve). Five (41.7%) patients were diabetics; the mean ultrafiltration volume was 1.86 ± 0.4 6L (Table 1). Treatments were completed without hypotension and without gastro-intestinal symptoms. Individual measurements obtained on separate study days were highly correlated with a negligible variation thus revealing a good reproducibility of the measurements. For example, flow index measurements repeated in subsequent weeks were highly correlated (r = 0.94, p<0.0001) and not different between studies done in the same patient. Hemodynamic variables measured in subsequent treatments done in the same patient were therefore averaged and used for further analysis. As expected, hematocrit increased from 35.13 ± 2.13 at *t_1_* to 36.72 ± 2.01% at *t_4_* (*p* = 0.003), paralleled by a decrease in plasma volume from 3.65 ± 0.77 to 3.40 ± 0.78 L (*p* = 0.008). Heart rate and mean arterial pressure did not change during the study period. All study subjects exhibited an ICG-PDR of larger than 10% (Table 2), a prerequisite for a valid estimate of hepato-splachnic blood flow *(Q)* by ICG-Clearance [4]. In the study population as a whole, hepato-splanchnic blood flow index (*QI*) dropped from 0.61 ± 0.22 at *t_1_* to 0.52 ± 0.20 L/min/m^2^ at *t_4_* (*p* = 0.003, Table 2), corroborated by a decrement of ICG disappearance rate from 19.3 ± 6.2 to 18.2 ± 5.2%/min (*p* = 0.001). Hepato-splanchnic vascular resistance index (*RI*) increased from 12.40 ± 4.21 at *t_1_* to 14.94 ± 6.36 *PRU*∙m² at *t_4_* (*p* < 0.001, Table 2), corresponding to a relative increase by 19.2% (*p* = 0.01, Table 3). There was no correlation between ultrafiltration rate and either *R* or *QI (*data not shown). The comparison of flow to resistance confirmed the expected hyperbolic relationship for constant mean arterial pressure with flow decreasing as resistance increased (Fig 1, top panel). Transformation of resistance (*RI*) into conductance (1/*RI*) provided the expected linear relationship with flow increasing as conductance increased (Fig 1, bottom panel). Notice that diabetic patients are clustered at the high resistance, low conductance end of these graphs.

**Table 1:** Patient and treatment characteristics.

	**All (*n* = 12)**	**Diabetics (*n* = 5)**	**Non-Diabetics (*n* = 7)**	**p**
**Female (n)**	6	4	2	
**Age (y)**	53 (32–80)	54 (47–80)	53 (32–71)	0.19
***M* (kg)**	71.9 ± 14.5	63.4 ± 10.3	78.0 ± 14.5	0.08
**Height (cm)**	170.3 ± 8.9	165.2 ± 10.0	174.0 ± 6.6	0.09
***BMI* (kg/m^2^)**	24.7 ± 3.9	23.1 ± 2.4	25.7 ± 4.6	0.27
***A* (m^2^)**	1.8 ± 0.2	1.7 ± 0.2	1.9 ± 0.2	0.06
**Dialysis vintage (mo)**	82.0 ± 60.5	77.8 ± 65.8	85.0± 61.5	0.85
***V*_uf_ (L)**	1.86 ± 0.46	1.85 ± 0.53	1.87 ± 0.44	0.95
**Na^+^_d_ (mmol/L)**	137.9 ± 2.6	138.3 ± 1.6	137.6 ± 3.3	0.66
***Q*_b_ (mL/min)**	288 ± 20	278 ± 18	294 ± 19	0.17
**HDF (n)**	10	3	7	
**HD (n)**	2	2	0	
**ACE-Inhibitor**	1	1	0	
**ß-blocker**	5	2	3	

*n*: number of subjects; *p*: probability; *M*: body mass at dry weight; *BMI*: body mass index; *A*: body surface area; *V*_uf_: ultrafiltration volume; Na^+^_d_ dialysate sodium concentration; *Q*_b_: extracorporeal blood flow; HDF: hemodiafiltration: HD: hemodialysis; ACE: angiotensin-converting-enzyme

**Table 2:** Hemodynamic variables at time points *t*_1_ through *t*_4_.

	**All (*n* = 12)**	**Diabetics (*n* = 5)**	**Non-Diabetics (*n* = 7)**	***p*^+^**
***MAP* (mmHg)**				
***t*_1_**	114 .2 ± 16.8	116.0 ± 3.4	112.9 ± 22.4	0.77
***t*_2_**	110.4 ± 16.9	114.9 ± 9.2	107.2 ± 20.9	0.46
***t*_3_**	109.7 ± 23.1	115.6 ± 26.4	105.5 ± 21.5	0.48
***t*_4_**	111.7 ± 22.4	116.8 ± 18.9	108.1 ± 25.4	0.53
p*	0.71	0.99	0.14	
***HR* (1/min)**				
***t*_1_**	70.5 ± 11.4	65.5 ± 14.5	74.0 ± 7.9	0.22
***t*_2_**	69.6 ± 12.9	64.7 ± 14.7	73.2 ± 11.3	0.28
***t*_3_**	69.3 ± 13.3	64.0 ± 18.7	73.1 ± 7.3	0.26
***t*_4_**	72.1 ± 15.2	66.2 ± 22.1	76.3 ± 6.7	0.28
p*	0.82	0.89	0.49	
***Hct* (%)**				
***t*_1_**	35.13 ± 2.13	35.73 ± 2.51	34.70 ± 1.90	0.44
***t*_2_**	35.67 ± 2.18	36.29 ± 2.70	35.22 ± 1.81	0.43
***t*_3_**	35.62 ± 1.83	35.52 ± 2.40	35.69 ± 1.51	0.88
***t*_4_**	36.72 ± 2.01	37.57 ± 2.38	36.11 ± 1.62	0.23
p*	0.003	0.17	*<* 0.001	
***V_p_* (L)**				
***t*_1_**	3.65 ± 0.77	3.03 ± 0.82	4.09 ± 0.29	0.009
***t*_2_**	3.48 ± 0.81	2.99 ± 0.85	3.84 ± 0.62	0.07
***t*_3_**	3.55 ± 0.83	2.96 ± 0.85	3.98 ± 0.52	0.03
***t*_4_**	3.40 ± 0.78	2.86 ± 0.82	3.79 ± 0.49	0.03
p*	0.008	0.03	0.03	
***QI* (L/min/m^2^)**				
***t*_1_**	0.61 ± 0.22	0.47 ± 0.13	0.72 ± 0.23	0.06
***t*_2_**	0.56 ± 0.19	0.43 ± 0.15	0.66 ± 0.18	0.04
***t*_3_**	0.54 ± 0.22	0.39 ± 0.14	0.64 ± 0.21	0.04
***t*_4_**	0.52 ± 0.20	0.39 ± 0.15	0.61 ± 0.19	0.07
p*	0.003	0.001	*p =* 0.07	
***ICG-DR* (%/min)**				
***t*_1_**	19.3 ± 6.2	16.2 ± 4.2	21.5 ± 6.6	0.15
***t*_2_**	18.8 ± 5.8	15.8 ± 4.2	20.9 ± 6.0	0.14
***t*_3_**	18.3 ± 5.5	15.7 ± 4.2	20.2 ± 5.8	0.17
***t*_4_**	18.2 ± 5.2	15.8 ± 4.5	19.9 ± 5.3	0.19
p*	0.001	0.29	0.002	
***RI* (mmHg∙s∙m^2^/mL)**				
***t*_1_**	12.40 ± 4.21	15.71 ± 4.23	10.03 ± 2.21	0.01
***t*_2_**	13.30 ± 5.43	17.64 ± 5.85	10.19 ± 2.06	0.01
***t*_3_**	14.27 ± 6.18	19.65 ± 5.99	10.43 ± 2.19	0.004
***t*_4_**	14.94 ± 6.36	20.29 ± 6.46	11.11 ± 2.29	0.005
p*	0.001	0.01	0.18	

*n*: number of subjects; *MAP*: mean arterial pressure; *HR*: heart rate: *Hct*: hematocrit: *V_p_*: plasma volume: *QI*: hepato-splanchnic blood flow index: *ICG-DR*, Indocyanine green disappearance rate: *RI*, hepato-splanchnic vascular resistance index; p*: probability for single factor variance analyses with repeated measurement; *p* ^+^: probability for two sample *t-*test for comparison between diabetics and non-diabetics

**Table 3:** Relative perfusion and resistance changes.

	**All (n = 12)**	**Diabetes (n = 5)**	**Non Diabetes (n = 7)**	***p*^+^**
***∆QI* (%)**				
***t*_2_**	-7.7 ± 8.1	-9.6 ± 7.5	-6.4 ± 8.8	0.52
***t*_3_**	-12.0 ± 11.3	-15.2 ± 10.7	-9.7 ± 11.9	0.44
***t*_4_**	-15.1 ± 10.5	-16.8 ± 13.7	-13.9 ± 8.5	0.67
p*	0.11	0.08	0.38	
***∆RI* (%)**				
***t*_2_**	6.1 ± 11.9	11.2 ± 13.2	2.5 ± 10.3	0.22
***t*_3_**	13.9 ± 20.9	26.3 ± 23.2	5.1 ± 14.9	0.08
***t*_4_**	19.2 ± 18.8	29.6 ± 21.3	11.8 ± 13.9	0.11
p*	0.01	0.02	0.29	

∆*QI*: change of hepato-splanchnic blood flow index at times *t*_2_, *t*_3_, and *t*_4_ relative to baseline at *t_1_*; ∆*RI*: change of hepato-splanchnic vascular resistance index at times *t*_2_, *t*_3_, and *t*_4_ relative to baseline at *t_1_*; p*: probability for single factor variance analyses with repeated measurement; *p* ^+^: probability for two-sample *t-*test for comparison between diabetics and non-diabetics

### Diabetic and non-diabetic subgroups

**Fig 2:**
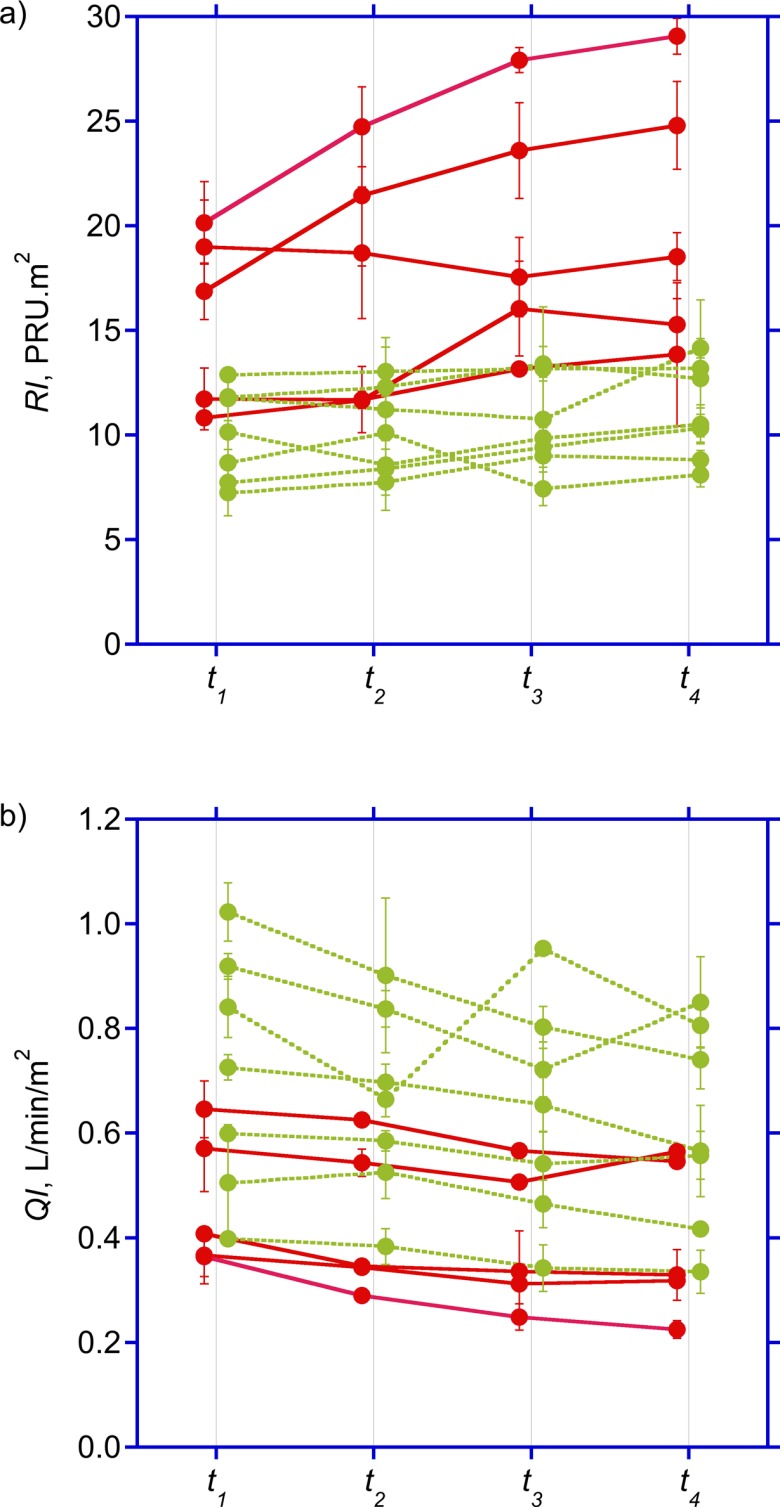
Time course of hepato-splanchnic hemodynamics. Time course of splanchnic vascular resistance index (*RI*) (top panel) and hepato-splanchnic perfusion index (*QI*) (bottom panel) measured at times *t*_1_ through *t*_4_ during dialysis in diabetic (red symbols) and non-diabetic (green symbols) subjects. Symbols represent average values ± standard deviations of duplicate measurements obtained in treatments separated by one week. For clarity, symbols for diabetics and non-diabetics are placed with a small left or right offset from the actual measuring times.

Diabetics and non-diabetics were comparable with respect to baseline anthropometric characteristics, dialysis vintage and ultrafiltration volumes (Table 1). Although both groups remained hemodynamically stable throughout the study period, hepato-splanchnic blood flow was significantly lower in diabetics than in the non-diabetic cohort at *t_2_* and *t_3_*, barely missing significance at *t_1_* and *t_4_* (Table 2, Fig 2, top panel). By contrast, both groups did not differ with regard to their ICG disappearance rate. The reduced hepato-splanchnic blood flow index in the diabetic subgroup was related to a significantly greater hepato-splanchnic vascular resistance index (*RI*) in this cohort (Table 2, Fig 2, bottom panel). The separation of groups is also clearly seen in the perfusion vs. resistance or conductance plots. Contrary to the disparities between absolute values of *QI* and *RI* at the different time points, we observed no significant difference in the relative changes of *QI* and *RI* between the two groups during HD (Table 3). Original data are provided in the supplementary file (S1 Table).

## Discussion

The main observation of this study is that hepato-splanchnic resistance was about 30% higher in diabetic compared to non-diabetic patients already at the beginning of measurements, 15 min into dialysis, corresponding to a hepato-splanchnic blood flow that was only two thirds of that in non-diabetic patients. Furthermore, to maintain a stable arterial blood pressure during ultrafiltration-induced decline in plasma volume hepato-splanchnic resistance increased during HD in non-diabetic as well as in diabetic patients. Interestingly, this increase was much more pronounced in diabetic subjects compared to non-diabetic subjects.

In our study population arterial pressure and heart rate remained unchanged throughout dialysis in spite of significant hemoconcentration and plasma volume reduction. These observations are akin with previous findings regarding hepato-splanchnic blood flow [4] [20]. The splanchnic vascular bed is characterized by high vascular capacitance and compliance and plays an important role to compensate for a fall in blood volume, venous return, central venous pressure, cardiac output, and arterial blood pressure. During ultrafiltration fluid is first removed from the blood volume and then partially refilled from the interstitial space [21]. The initial removal is primarily from the compliant venous part of the circulation thereby causing a small decrease in venous pressures [22]. The resulting drop in central venous pressure will therefore also tend to lower hepatic venous pressure. As central venous or hepatic venous pressures were not measured in this study, the true arterio-venous pressure drop may have been underestimated, resulting in a small underestimation of hepato-splanchnic resistance, if at all. Therefore, the increase in hepato-splanchnic resistance calculated in this study represents a conservative estimate given the uncertainties of true hepatic venous pressure changes.

**Fig 3:**
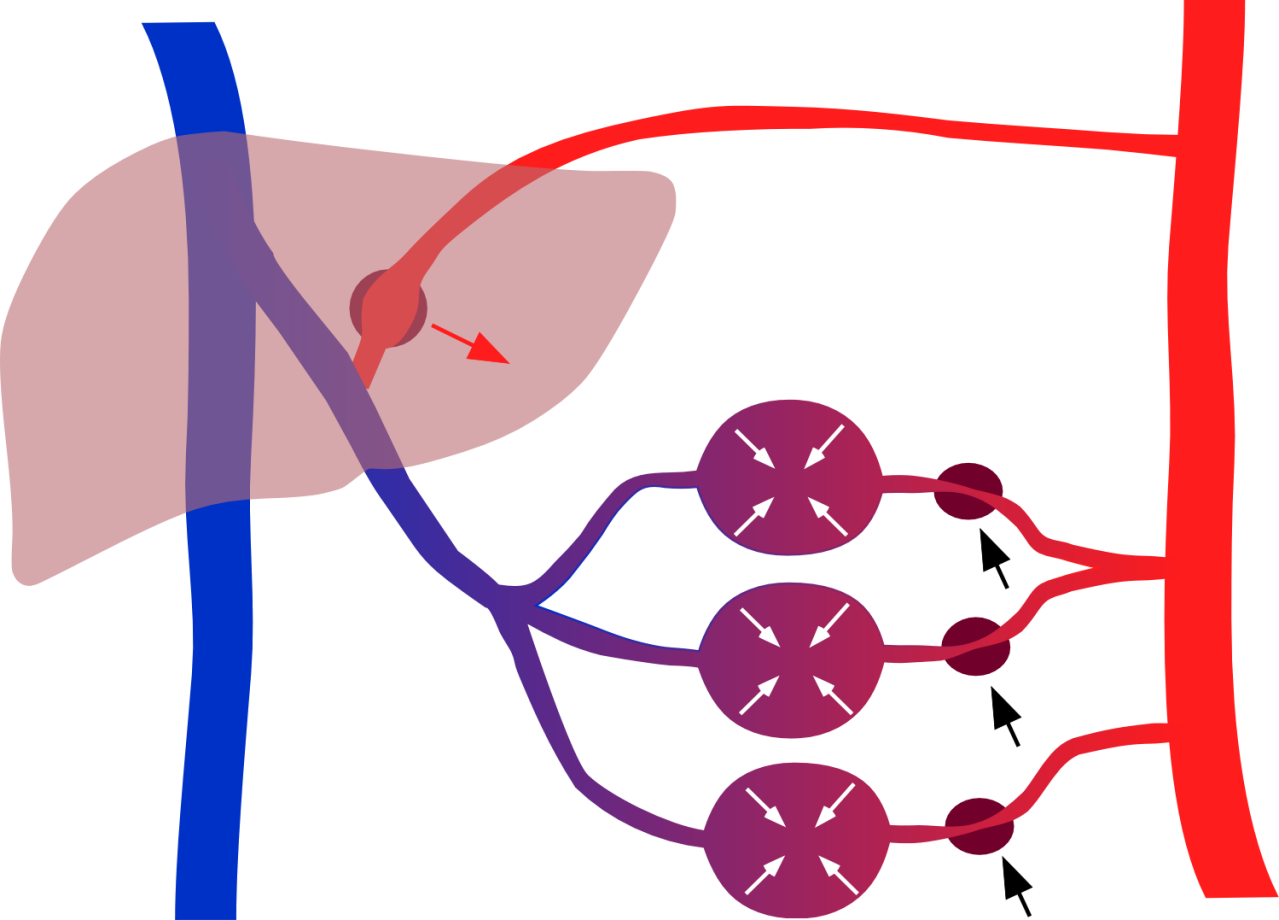
Scheme of hepato-splanchinc contribution for hemodynamic stability. Splanchnic vasoconstriction reduces arterial inflow (black arrows) thereby lowering downstream distending pressures and mobilizing blood volume (white arrows) sequestered in the compliant splanchnic vasculature. At the same time reduced portal vein flow draining form splanchnic vascular beds (purple) causes a compensatory increase (red arrow) in the separate hepatic arterial blood flow (red) because of compensatory vasodilation in Mall´s space.

The hemodynamic effects of ultrafiltration in hemodialysis have been reviewed elsewhere [23]. Briefly, the decrease in venous pressure is assumed to reduce the ventricular preload thereby reducing cardiac output and arterial pressures. This fall will then be compensated by the baroreflex mechanism through central and peripheral control actions. The resulting increase in peripheral resistance not only compensates for the decrease in arterial pressures but also reduces the downstream distending pressures in compliant vascular beds such as the hepato-splanchnic circulation, thereby shifting blood to central parts of the circulation compensating for the initial decrease in central venous pressure and right atrial pressure (Fig 3). This volume shift from a compliant vascular bed is also known as DeJager-Krogh effect [24]. In our study the effect of ultrafiltration on splanchnic vasoconstriction is documented by a clear reduction in ICG clearance. Our data indicate that hemodynamic stability is achieved at the expense of a significantly reduced hepato-splanchnic perfusion. It is possible that a decrease of hepato-splanchnic perfusion causes critical intestinal ischemia, especially during 4 hours of HD and ultrafiltration although we cannot provide direct evidence for this phenomenon in our study. Moreover, it is currently not possible to predict at what level of hepato-splanchnic vasoconstriction a critical intestinal ischemia is likely to occur. However, diabetic patients starting with a low perfusion seem to be at special risk. In this scenario, however, the liver itself is protected against hypoperfusion because of its dual blood supply and the hepatic-arterial buffer response (HABR) [25] and a critical hepatic ischemia therefore appears to be less likely. The hepato-splanchinc flow measured in this study represents the sum of both portal venous and hepatic arterial inflows, and it is therefore not possible to allocate the overall reduction in hepato-splanchnic blood flow to a specific source of the dual blood supply (Fig 3). Moreover, because of hepatic-arterial buffer response intestinal perfusion might be reduced to a much larger extent than total hepato-splanchnic blood flow. As a consequence, bacterial translocation, endotoxemia, and systemic inflammation might develop as a result of an ischemic gut barrier during HD [5] [6] [26]. On the other hand, the ischemic splanchnic vascular bed may act as a culprit of intradialytic hypotension by itself. It has been proposed that the ischemic gut is an important source of adenosine, a purine nucleoside with vasodilating and cardiodepressant properties, rapidly leading to profound intradialytic hypotension [27] [28].

The underperfusion of the hepato-splanchnic vascular bed especially in diabetics is not easy to explain but it is in line with the occurrence of myocardial stunning observed shortly after starting HD [10] [29]. Since baseline measurements were obtained after having established the extracorporeal circulation the effect could be due to the mode of connecting the patient to the extracorporeal circulation where only part of the priming volume is infused whereas about 100 to 150 mL of priming volume are discarded leading to an abrupt blood volume drop of 2 to 3%. Diabetic patients could be more susceptible to this effect. The low perfusion and the high resistance of the splanchnic region measured already at treatment start indicate a reduced range to further increase splanchnic vascular resistance and compensate for UF-induced hypovolemia.

So far only a few studies addressed the impact of ultrafiltration on hepato-splanchnic resistance and blood flow during HD. In an early study the splanchnic content of ^99m^Tc-labeled erythrocytes decreased by 10% with accelerated ultrafiltration of 3.7 L during 2 h [3]. However, a change in volume cannot directly be translated into the same change in perfusion. A different group reported a 22% decrease of hepato-splanchnic blood flow during dialysis in acute kidney disease patients using constant ICG infusion given an UF-volume of 2 L [4]. The discrepancy between these studies and our data can be explained by different study settings such as including a heterogeneous ICU-cohort with acute renal failure in the latter study.

Our study encourages the assumption that diabetic dialysis patients might be particularly prone to critical splanchnic ischemia during UF. Interestingly, we did not observe any differences in the relative changes of hepato-splanchnic blood flow or hepato-splanchnic resistance between both groups. This suggests that vascular reactivity was maintained in diabetics. We can only speculate about the mediators of splanchnic vasoconstriction leading to the marked increase in vascular resistance from the onset of dialysis observed in our diabetic patients. Severe systemic atherosclerosis inherent to the diabetic HD-population might play an important role. However, diabetics represent only a small proportion of patients with chronic mesenteric ischemia in the general population [30], suggesting that other mechanisms are likely to be involved.

Elevated catecholamine levels capable of increasing the vascular tone in the splanchnic circulation are frequently found among diabetic ESKD-patients with diabetic neuropathy [31] [32] [33]. Another possible trigger of splanchnic vasoconstriction is the hypothalamic hormone arginine vasopressin (AVP). Apart from its role in osmoregulation it is an important systemic vasoconstrictor by activating vascular V_1_-receptors in skin, skeletal muscle and the splanchnic region [34] [35]. Studies revealed that dialysis patients exhibit higher AVP levels than healthy individuals for reasons that are still under debate [33] [36] [37]. It has been shown that AVP is important in maintaining hemodynamic stability during HD [38], and it has been speculated whether an inadequate rise of AVP contributes to the development of intradialytic hypotension [37] [39] [40]. Furthermore, it has been shown that in the presence of diabetic neuropathy, a condition where other neurohumoral pressor systems are compromised, AVP becomes essential in maintaining blood pressure during circulatory stress [41]. All these findings suggest that humoral factors are responsible for the altered vascular response of the hepato-splanchnic region in diabetics compared to non-diabetic patients.

In critical care medicine, the ICG disappearance rate is frequently determined as a marker of hepatic function and a surrogate of hepato-splanchnic blood flow based on its good clinical applicability [42] [43]. In our study, however, the aforementioned discrepancies in hepato-splanchnic perfusion could only be recognized by determining ICG clearance. There are important differences with regard to the measurement and interpretation of ICG elimination. For example, in the constant infusion approach the infusion rate (*Q_inf_*) to maintain a constant ICG concentration (*c_b_*) in arterial or mixed venous blood is used to calculate clearance (*K*) as the ratio of *Q_inf_* to *c_b_* (*K* = *Q*_inf_/*c*_b_). This, however, requires a constant plasma volume and a steady state distribution of red blood cells [44] analyzed in a companion paper [45]. In the bolus approach the elimination of ICG produces an exponential decline of ICG blood (or plasma) concentrations. Log transformation of concentrations produces a linear decline, and the rate constant (*k*, in 1/min) is determined by the slope of this line. The rate constant quantifies the disappearance rate (in 1/min or %/min) and the half-life (in min), but it is insufficient to quantify hepatic clearance (*K*) and hepato-splanchnic blood flow (*Q*) as the measurement of clearance (*K = k∙V*) also requires quantitative information on the distribution volume (*V*). Non-invasive devices used for measurement of ICG disappearance rate only report the elimination rate constant, which provides useful information on hepatic function, but fail to measure hepatic clearance or hepato-splanchnic blood flow. This discrepancy is clearly seen in our data where elimination rate remained almost unchanged without differences between diabetics and non-diabetics (Tab. 2), but where hepato-splanchnic blood flow was lower in diabetics and significantly decreased during HD and UF (Fig 2, bottom panel).

A limitation of our study is the relatively small number of patients which is not unusual for this type of research [3, 4]. Moreover, all studies were repeated in subsequent midweek treatments and showed a high reproducibility with negligible variation, indicating the validity of our measurements. A further limitation is that we cannot provide information on the exact fraction of cardiac output delivered to the hepato-splanchnic circulation received, and how this fraction changed during ultrafiltration. Furthermore we did not take serial blood samples to examine whether the decrease in hepato-splanchnic blood flow resulted in an increased risk for bacterial translocation, endotoxemia and systemic inflammation. Therefore we cannot provide evidence for a causal relationship between reduced hepato-splanchnic blood flow, intestinal ischemia, and the occurrence of endotoxemia and systemic inflammation.

In conclusion, this study reveals that in chronic HD patients even a modest UF volume causes a considerable increase in hepato-splanchnic vascular resistance due to active vasoconstriction, especially in diabetics. To the best of our knowledge this is the first study to show that diabetic HD patients exhibit a particularly high hepato-splanchnic resistance and a reduced hepato-splanchnic blood flow. It can be speculated that diabetics therefore incur a higher risk for complications such as endotoxemia, systemic inflammation, and intradialytic hypotension. Further studies are needed to provide direct evidence for a causal relationship between ultrafiltration induced splanchnic ischemia and the occurrence of endotoxemia and systemic inflammation.

## Supporting Information

S1 Table Original data. Original data of individual measurements (*n* = 24) obtained in 12 subjects in two subsequent studies. Abbreviations: *F*: female; *M*: male; *DM*: diabetic; *ND*: non-diabetic; *BMI*: body mass index; *BSA*: body surface area; *V*_uf_: ultrafiltration volume; *MAP*: mean arterial pressure; *HR*: heart rate: *Hct*: hematocrit: *V*_*p*_: plasma volume: *QI*: hepato-splanchnic blood flow index: *ICG-DR*, Indocyanine green disappearance rate: *RI*, hepato-splanchnic vascular resistance index; numbers behind variables refer to measuring times *t*.
(XLSX)
